# Tfap2a Promotes Specification and Maturation of Neurons in the Inner Ear through Modulation of Bmp, Fgf and Notch Signaling

**DOI:** 10.1371/journal.pgen.1005037

**Published:** 2015-03-17

**Authors:** Husniye Kantarci, Renee K. Edlund, Andrew K. Groves, Bruce B. Riley

**Affiliations:** 1 Biology Department, Texas A&M University, College Station, Texas, United States of America; 2 Program in Developmental Biology, Baylor College of Medicine, Houston, Texas, United States of America; 3 Department of Molecular and Human Genetics, Baylor College of Medicine, Houston, Texas, United States of America; 4 Department of Neuroscience, Baylor College of Medicine, Houston, Texas, United States of America; University of Pennsylvania School of Medicine, UNITED STATES

## Abstract

Neurons of the statoacoustic ganglion (SAG) transmit auditory and vestibular information from the inner ear to the hindbrain. SAG neuroblasts originate in the floor of the otic vesicle. New neuroblasts soon delaminate and migrate towards the hindbrain while continuing to proliferate, a phase known as transit amplification. SAG cells eventually come to rest between the ear and hindbrain before terminally differentiating. Regulation of these events is only partially understood. Fgf initiates neuroblast specification within the ear. Subsequently, Fgf secreted by mature SAG neurons exceeds a maximum threshold, serving to terminate specification and delay maturation of transit-amplifying cells. Notch signaling also limits SAG development, but how it is coordinated with Fgf is unknown. Here we show that transcription factor Tfap2a coordinates multiple signaling pathways to promote neurogenesis in the zebrafish inner ear. In both zebrafish and chick, Tfap2a is expressed in a ventrolateral domain of the otic vesicle that includes neurogenic precursors. Functional studies were conducted in zebrafish. Loss of Tfap2a elevated Fgf and Notch signaling, thereby inhibiting SAG specification and slowing maturation of transit-amplifying cells. Conversely, overexpression of Tfap2a inhibited Fgf and Notch signaling, leading to excess and accelerated SAG production. However, most SAG neurons produced by Tfap2a overexpression died soon after maturation. Directly blocking either Fgf or Notch caused less dramatic acceleration of SAG development without neuronal death, whereas blocking both pathways mimicked all observed effects of Tfap2a overexpression, including apoptosis of mature neurons. Analysis of genetic mosaics showed that Tfap2a acts non-autonomously to inhibit Fgf. This led to the discovery that Tfap2a activates expression of Bmp7a, which in turn inhibits both Fgf and Notch signaling. Blocking Bmp signaling reversed the effects of overexpressing Tfap2a. Together, these data support a model in which Tfap2a, acting through Bmp7a, modulates Fgf and Notch signaling to control the duration, amount and speed of SAG neural development.

## Author Summary

Neurons of the statoacoustic ganglion (SAG) transmit impulses from the inner ear necessary for hearing and balance. SAG cells exhibit a complex pattern of development, regulation of which remains poorly understood. Here we show that transcription factor Tfap2a coordinates multiple cell signaling pathways needed to regulate the quantity and pace of SAG neuron production. SAG progenitors originate within the developing inner ear and then migrate out of the ear towards the hindbrain before forming mature neurons. We showed previously that Fgf initiates formation of SAG progenitors in the inner ear, but rising levels of Fgf signaling eventually terminate this process. Elevated Fgf also stimulates proliferation of SAG progenitors outside the ear and delays their maturation. Notch signaling is also known to limit SAG development. Tfap2a governs the strength of Fgf and Notch signaling by activating expression of Bmp7a, which inhibits Fgf and Notch. Together these signals stabilize the pool of SAG progenitors outside the ear by equalizing rates of maturation and proliferation. This balance is critical for sustained accumulation of SAG neurons during larval growth as well as regeneration following neural damage. These findings could inform development of stem cell therapies to correct auditory neuropathies in humans.

## Introduction

Vestibular and auditory information is transmitted from the inner ear to the hindbrain via neurons of VIII.th cranial ganglion, also known as the stato-acoustic ganglion (SAG). SAG neurons are formed by a complex but poorly understood multi-step process that begins in the otic vesicle, the precursor of the inner ear. In the first step, SAG neuroblasts are specified in the floor of the otic vesicle and are marked by the expression of proneural gene *neurogenin1* (*ngn1*) [1,2]. After specification, neuroblasts delaminate from the otic epithelium via epithelial-mesenchymal transition and migrate to a region between the otic vesicle and hindbrain. In zebrafish, markers of later stages of differentiation are usually not expressed within the otic epithelium. Upon delamination, however, neuroblasts quickly lose expression of *ngn1* and upregulate the related factor *neurod* [1,3]. *neurod-*expressing cells form a population of migrating and proliferating precursors called the transit-amplifying (TA) pool [4]. As cells in the TA pool differentiate into mature neurons they lose expression of *neurod* and upregulate early neuronal markers *islet1* and *islet2b* [5]. Newly formed neurons extend processes bi-directionally to connect sensory epithelia with central targets in the hindbrain. SAG development in chick and mouse embryos follows a similar course except that transit-amplification and expression of *NeuroD* and *Isl1/2* begin while neuroblasts still reside within the otic epithelium [2,6,7].

We previously showed that Fgf signaling regulates each step in SAG development in zebrafish [5]. Specification of SAG neuroblasts is initiated by a low level of Fgf signaling. As SAG neurons mature they begin to express *fgf5* such that rising levels of Fgf eventually become inhibitory to *ngn*1 expression in the otic vesicle. Consequently, neuroblast specification starts to decline after 24 hpf and ceases entirely by 42 hpf [5,8]. Elevated Fgf also delays terminal differentiation of cells in the TA pool. The TA pool is thereby maintained as a relatively stable population in which the rate of proliferation closely matches the rate of terminal differentiation.

The otic vesicle originates from an ectodermal thickening called the otic placode. The otic placode, along with all other cranial placodes, emerges from a contiguous region of pre-placodal ectoderm (PPE) that forms around the anterior neural plate by the end of gastrulation [9,10]. In zebrafish competence to form PPE is regulated by four transcription factors: Tfap2a, Tfap2c, Gata3 and Foxi1 [9,11]. These transcription factors are also essential for later development of a subset of cranial placodes, including the otic placode. For example, in response to inductive Fgf signaling *foxi1* expression upregulates in nascent otic/epibranchial placodes; and disruption of *foxi1* leads to severe deficiencies of epibranchial and otic tissue in zebrafish [12–14]. A similar role has been shown recently for *Foxi3* in mouse and chick [15,16]. Expression of *gata3* also regulates otic development, becoming localized to the nascent otic placode [17] and to discrete regions of the otic vesicle [18–20]. Disruption of *Gata3* in mouse causes severe defects in otic vesicle development [18], including deficiencies of sensory epithelia and improper wiring of auditory neurons [21–23]. In contrast, less is known about later roles of *tfap2a/c*. *tfap2a* is best known for its role in the early differentiation and survival of neural crest cells [24–32] and together with *tfap2c* is indispensible for neural crest specification in zebrafish [26,28]. However, whether *tfap2a/c* genes regulate later development in the otic placode and vesicle has not been investigated.

Here we report that *tfap2a* is expressed throughout the nascent otic placode and is later restricted to a ventrolateral region in the otic vesicle overlapping with the neurogenic domain. Misexpression of *tfap2a* leads to excess specification and precocious differentiation of SAG neurons whereas knockdown of *tfap2a* causes reduced neurogenesis and delayed differentiation of SAG precursors. Further investigation revealed that *tfap2a* acts non-autonomously through upregulation of *bmp7a*, which in turn restricts Fgf and Notch signaling to promote specification and differentiation of SAG precursors.

## Results

### Expression of *tfap2a* during otic development

**Fig 1:**
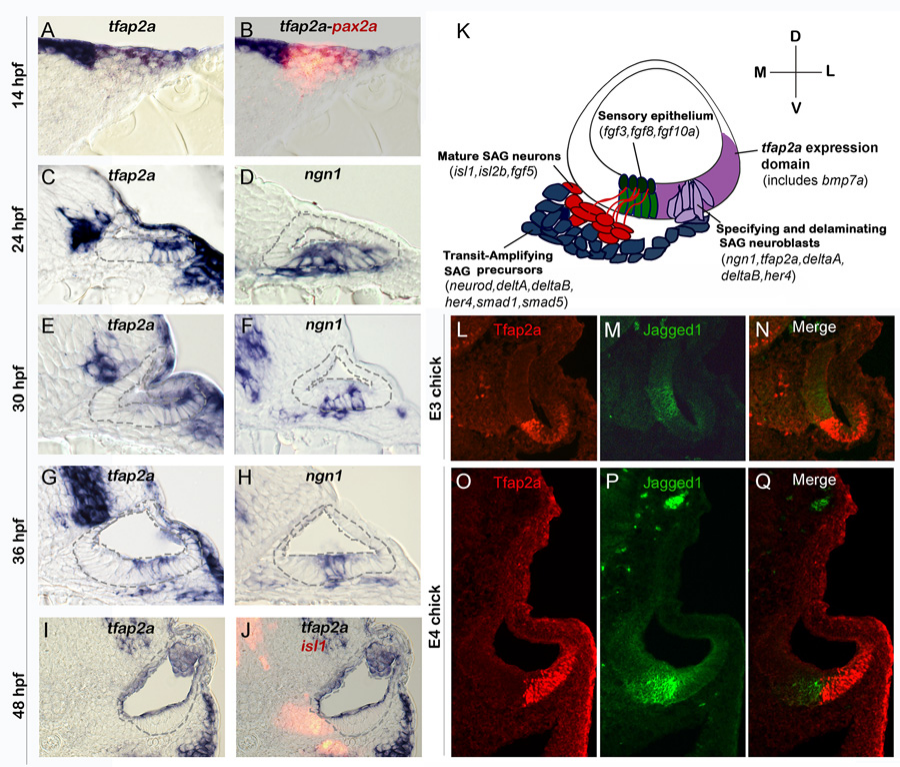
Conserved expression of *tfap2a* during otic neurogenesis. All images show cross-sections of the otic placode or vesicle in wild type zebrafish embryos (A-J) or chick embryos (L-Q) with a dorsal up and medial to the left. (A, B) At 14 hpf (10 somites) *pax2a* (red) marks the precursor cells in the emerging otic placode that are co-labeled with *tfap2a* (blue). (C-H) Cross-sections through the widest part of the neurogenic domain of the otic vesicle, just posterior to the utricular macula. The outer and inner edges of the otic vesicle are outlined. Patterns of *ngn1* or *tfap2a* are shown at the indicated times. *tfap2a* is expressed in the ventrolateral part of the otic vesicle, which partially overlaps the domain of *ngn1* expression. (I-J) Cross-sections passing through the utricular macula of specimens co-stained for Isl1 (red) and *tfap2a* (blue) at 48 hpf. Expression of *tfap2a* is not detected in the floor of the otic vesicle or in the mature SAG neurons at this time. (K) Schematic summary of SAG development in zebrafish, including regional markers. Neuroblasts are specified and delaminate from the otic vesicle (light purple) adjacent to nascent sensory epithelia (green). Recently delaminated neuroblasts migrate towards hindbrain and continue to proliferate, forming the transit-amplifying pool (blue). Neuroblasts then stop dividing and differentiate into mature neurons (red). Relevant genes expressed in each domain are indicated. Expression of *tfap2a* (dark purple) overlaps the neurogenic domain, as well as the domain of *bmp7a* expression. Note that all of the tissues indicated express Fgf-target genes (*etv5b* and *spry4*) and transducers of Bmp (*smad1* and *smad5*), but transit amplifying SAG precursors show specific upregulation of *smad1* and *smad5* [37]. (L-Q) Cross-sections through the otic vesicle of chick embryos at days 3 and 4 (E3 and E4). The sensory region is labeled with Jagged-1 (green). Tfap2a (red) is expressed in the ventrolateral otic domain in chick embryos similar to the pattern observed in zebrafish.

To begin to assess potential functions of *tfap2a* in otic development, we examined expression of *tfap2a* in the otic placode and early otic vesicle in zebrafish. At 14 hpf (10 somites) when otic cells first form a morphological placode, *tfap2a* is expressed broadly throughout the placode as shown by co-staining for the early otic marker *pax2a* (Fig. 1A-B). The level of *tfap2a* expression varies, with higher levels in dorsal and lateral otic cells. Otic expression in general appears much weaker than in surrounding neural crest cells. By 24 hpf, expression of *tfap2a* in the otic vesicle is restricted to ventrolateral cells. This domain partially overlaps with the neurogenic domain of the otic vesicle, marked by the proneural gene *ngn1* (Fig. 1C-D). Neurogenesis declines sharply by 30 hpf and ceases entirely by 42 hpf [5,8]. Similarly, the level of *tfap2a* gradually declines in the neurogenic domain after 30 hpf and is no longer detectable by 48 hpf (Fig. 1E-I). Despite initial expression of *tfap2a* in at least some neuroblasts in the otic vesicle, expression is lost in most neural precursors as they delaminate from the otic vesicle. Mature neurons of the statoacoustic ganglion (SAG), marked by expression of Isl1, show no detectable expression of *tfap2a* (Fig. 1J). These data are consistent with the possibility that *tfap2a* is involved in at least some aspects of neurogenesis in the otic vesicle. Expression patterns of *tfap2a*, *ngn1*, and other key genes involved in SAG development are summarized in Fig. 1K.

To examine the possibility that expression is conserved in amniote vertebrates, we examined expression of Tfap2a in chick embryos. In agreement with the patterns observed in zebrafish, chick embryos also show expression in ventrolateral regions of the otic vesicle (Fig. 1L-Q). Moreover, in chick as in zebrafish, the domain of Tfap2a expression abuts the sensory domain with little or no overlap. The similar expression patterns seen in zebrafish and chick potentially reflect a broadly conserved role in early otic development.

### Effects of Tfap2a on neurogenesis in the otic vesicle

**Fig 2:**
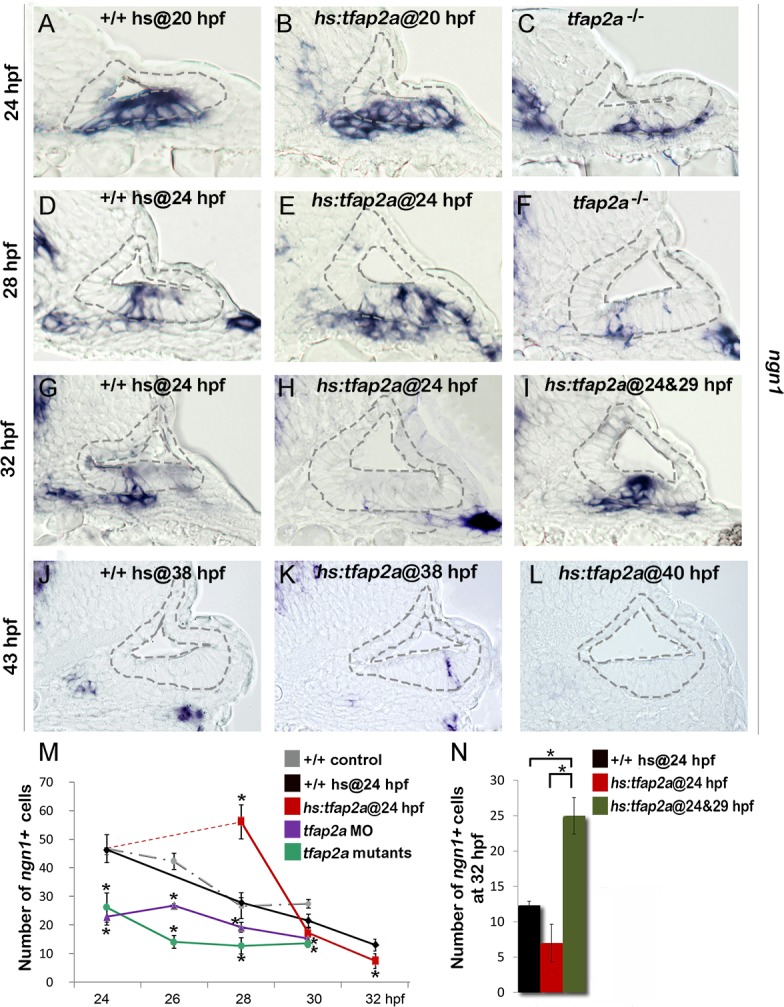
Tfap2a enhances otic neurogenesis. (A-L) Cross-sections (medial left, dorsal up) through the otic region just posterior to the utricular macula showing *ngn1* expression in +/+ control embryos, *hs*:*tfap2a* embryos and *tfap2a* ^*-/-*^ mutants at the indicated times. Wild-type and *hs*:*tfap2a* embryos were heat shocked as indicated in each panel. Overexpression of *tfap2a* increases the number of neuroblasts in the otic vesicle whereas loss of *tfap2a* slows down and decreases otic neurogenesis. The outer and inner edges of the otic vesicle are outlined in each image. (M, N) Mean and standard deviation of the total number of *ngn1* positive cells in the otic epithelium from 24 to 32 hpf for the genotypes indicated in the color key (counted from serial sections, n = 3–7 ears per time point). Asterisks (*) indicate statistically significant differences between groups indicated by brackets (N) or compared to control embryos (M).

To explore the role of *tfap2a*, we characterized the effects of *tfap2a* loss of function or misexpression on neurogenesis in the otic vesicle in zebrafish. Disruption of *tfap2a* causes no overt defects in morphogenesis of the otic vesicle [27]. However, *tfap2a* ^*-/-*^ (*lockjaw*) mutants produced only half the normal number of *ngn1*-positive neuroblasts in the otic epithelium at 24 hpf, the stage when neurogenesis normally peaks in wild-type embryos (Fig. 2A, C). At later stages, too, *tfap2a* ^*-/-*^ mutants continued to show significant deficiencies in neuroblast specification (Fig. 2D, F, M). Similar results were seen in *tfap2a* morphants (*tfap2a*-MO, Fig. 2M). We next used a heat-shock inducible transgenic line, *hs*:*tfap2a*, to misexpress *tfap2a* at various developmental stages. Activation of *hs*:*tfap2a* at 20 hpf increased the peak number of neuroblasts in the otic vesicle at 24 hpf by 30% (58.33±3.21 *ngn1+* cells in *hs*:*tfap2a* embryos vs. 44.5±2.65 cells in controls, Fig. 2B). Activation of *hs*:*tfap2a* at 24 hpf prolonged the phase of peak neurogenesis, resulting in twice the normal number of neuroblasts at 28 hpf (Fig. 2D, E, M). Despite the initial surge, however, the number of *ngn1+* neuroblasts subsequently declined sharply in transgenic embryos, dropping below the level seen in control embryos at 30 hpf and thereafter (Fig. 2G, H, M). Because transgene activity decays 5 hours after heat shock (S1 Fig.), we tested the effects of serial heat shocks at 24 hpf and 29 hpf. This resulted in elevated neurogenesis through at least 32 hpf, when transgenic embryos had twice as many *ngn1+* cells in the otic epithelium as in control embryos (Fig. 2G, I, N). Neurogenesis in the ear normally ceases by 42 hpf [5,8], prompting us to investigate whether termination of neurogenesis could be altered by later misexpression of *tfap2a*. Indeed, activation of *hs*:*tfap2a* at 38 hpf prolonged specification of neural precursors, as *ngn1+* neuroblasts were still present in the otic vesicle through at least 43 hpf (Fig. 2J, K). In contrast, activation of *hs*:*tfap2a* at 40 hpf was not sufficient to prevent or delay the cessation of neurogenesis in the otic vesicle (Fig. 2L). Overall these results indicate that *tfap2a* enhances neurogenesis in the ear but cannot induce ectopic neurogenesis beyond the floor of the otic vesicle nor reactivate neurogenesis after it has stopped.

### Effects of Tfap2a on later stages of SAG development

**Fig 3:**
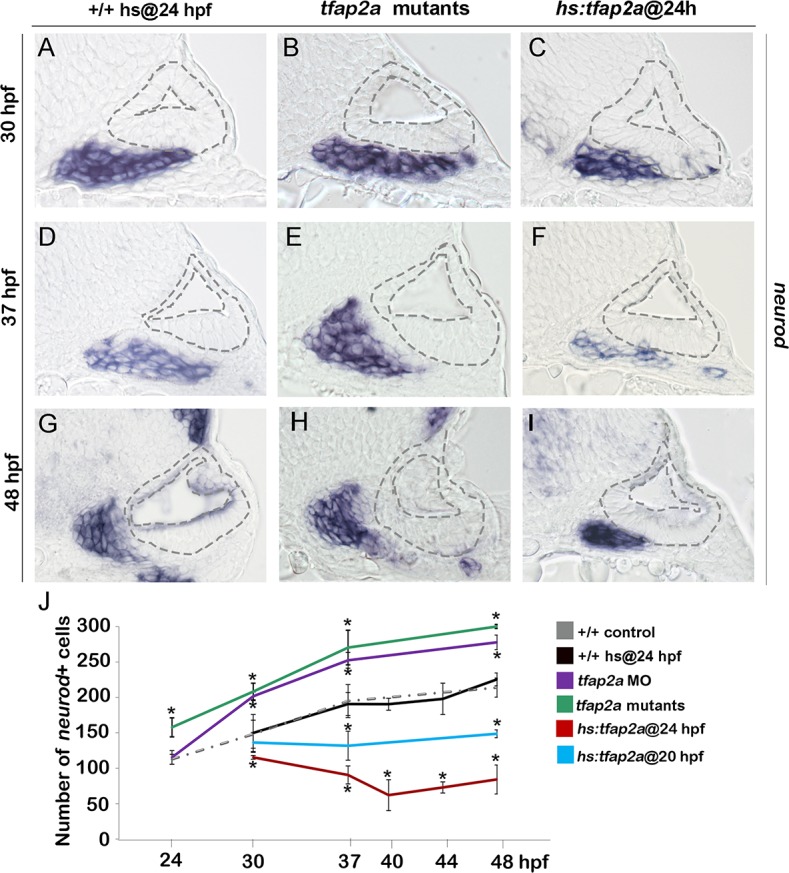
Tfap2a regulates the number of transit-amplifying SAG precursors. (A-I) Cross-sections (medial left, dorsal up) at the level of the utricular macula showing *neurod* expression in +/+ control, *tfap2a* mutants and *hs*:*tfap2a* embryos. Wild-type and *hs*:*tfap2a* embryos were heat shocked at 24 hpf. Disruption of *tfap2a* leads to accumulation of excess TA cells whereas *tfap2a* overexpression decreases the number of the TA cells. The outer and inner edges of the otic vesicle are outlined in each image. (J) Mean and standard deviation of the total number of *neurod* positive SAG precursors for the genotypes and conditions indicated in the color key (counted from serial sections, n = 3–6 ears per time point). Asterisks (*) indicate significant differences from control specimens.

**Fig 4:**
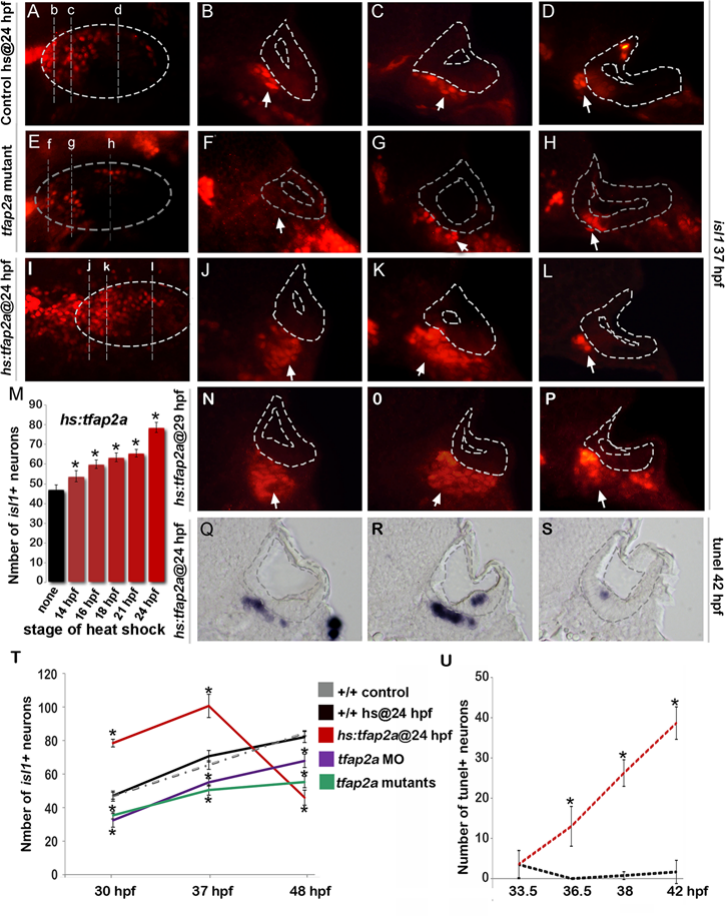
Tfap2a regulates maturation of SAG neurons. (A-L) Images of anti-Isl1 antibody staining in control embryos (A-D), *tfap2a* ^*-/-*^ mutants (E-H) and *hs*:*tfap2a* embryos (I-L) at 37 hpf. Control and *hs*:*tfap2a* embryos were heat shocked at 24 hpf. Whole-mount specimens (A, E, I) show dorsolateral (anterior to the left) and indicate planes of cross-section in (B-D), (F-H) and (J-L). Cross-sections are oriented with dorsal up and medial to the left. The otic vesicle is outlined in each image. White arrows indicate the SAG population to help distinguish it from other Isl1+ populations present in some sections. (M) Total number of Isl1+ neurons in *hs*:*tfap2a* embryos at 30 hpf following heat shock at the indicated times (n = 10–15 each). (N-P) Cross-sections of a *hs*:*tfap2a* specimen at 37 hpf following heat shock at 29 hpf. Planes of section are similar to those shown in (I). (Q-S) Cross-sections of a *hs*:*tfap2a* embryo stained for TUNEL positive SAG neurons at 42 hpf. (T) Mean and standard deviation of the total number of Isl1+ SAG neurons at the indicated times in +/+ embryos, *hs*:*tfap2a* embryos, *tfap2a* ^*-/-*^ mutants and *tfap2a* morphants (n = 7–34 embryos per time point). (U) Mean and standard deviation of the total number of TUNEL positive SAG neurons at the indicated times in control embryos and *hs*:*tfap2a* embryos (counted from serial sections, n = 3–6 ears per time point). Asterisks (*) indicate statistically significant differences compared to control embryos.

We next examined whether altered levels of neuroblast specification caused by manipulating *tfap2a* function were followed by changes in later stages of neuronal differentiation. Normally, newly specified neuroblasts delaminate from the otic vesicle, lose expression of *ngn1* and initiate expression of *neurod*, a marker of the “transit-amplifying” (TA) stage of development [1,5] (Fig. 1K). Surprisingly, despite reduced neurogenesis in *tfap2a* ^*-/-*^ mutants and *tfap2a* morphants, the number of *neurod*+ TA cells was greater than normal at every time point examined (Fig. 3B, E, H, J). Conversely, despite the large increase in neurogenic specification caused by overexpression of *tfap2a* at 24 hpf, the number of *neurod*-expressing TA cells was reduced at all time points through 48 hpf (Fig. 3C, F, I, J). We hypothesized that changes in the size of the TA pool reflect changes in the overall pace of neuronal differentiation. To test this we examined expression of Isl1, a marker of mature SAG neurons. We observed that *tfap2a* ^*-/-*^ mutants and *tfap2a* morphants produced fewer than normal neurons despite the increased number of *neurod*+ cells (Fig. 4E-H, T). The deficiency in neuronal maturation persisted in *tfap2a* ^*-/-*^ mutants through at least 72 hpf (S2 Fig.). In contrast, activation of *hs*:*tfap2a* had the opposite effect. Activation of *hs*:*tfap2a* during placodal stages elevated accumulation of Isl1+ neurons at 30 hpf, and the fold-stimulation was progressively increased with successively later stages of activation (Fig. 4M). Activation of *hs*:*tfap2a* at 24 hpf led to maximal accumulation of neurons, with nearly twice the normal number of Isl1+ neurons observed in transgenic embryos at 30 hpf and 37 hpf (Fig. 4I-L, M, T). Interestingly, transgene activation at these early stages enhanced accumulation of anterior (vestibular) SAG neurons but not posterior (auditory) neurons (Fig. 4D, L). However, activating *hs*:*tfap2a* expression at 29 hpf increased accumulation of both anterior and posterior neurons (Fig. 4N-P), consistent with our previous findings that auditory neurons are specified at later stages than vestibular neurons [5]. Together these data indicate that disruption of *tfap2a* inhibits neurogenesis and slows neural maturation, whereas misexpression of *tfap2a* stimulates neuroblast specification and accelerates subsequent differentiation.

Importantly, although activation of *hs*:*tfap2a* at 24 hpf led to elevated accumulation of mature neurons through 37 hpf, the number of mature neurons fell dramatically thereafter to roughly half normal by 48 hpf (Fig. 4T). This decline was preceded by a marked increase in the rate of apoptosis amongst mature neurons (Fig. 4Q-S, U). Elevated cell death possibly reflects insufficient duration of earlier stages of differentiation and consequent misregulation of factors required for neuronal survival (see below).

### Effects on patterning in the otic vesicle

**Fig 5:**
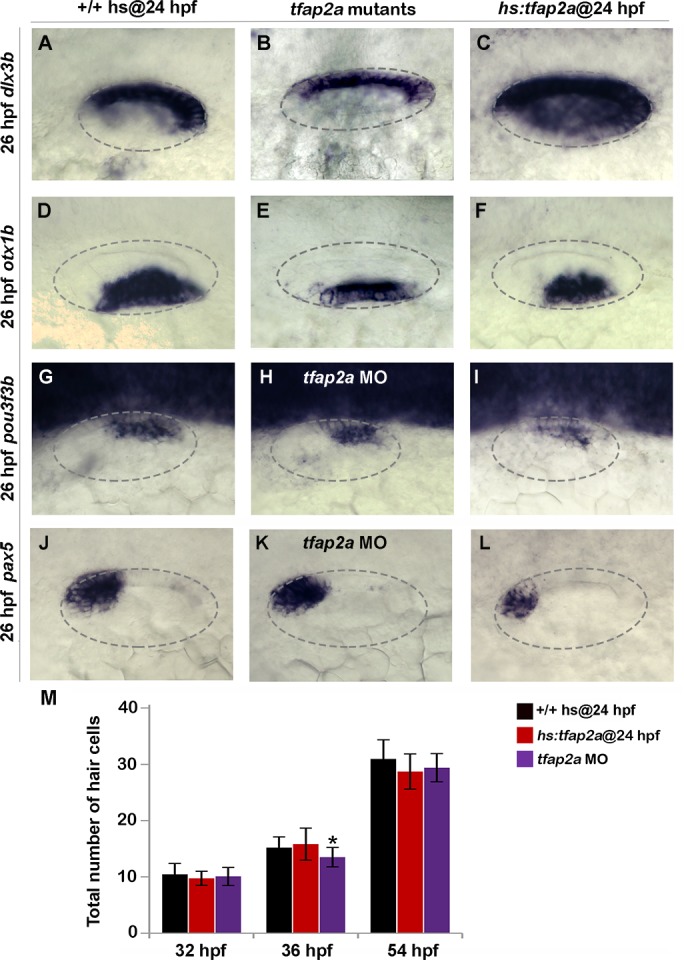
Effects of *tfap2a* knockdown and overexpression on otic vesicle patterning. (A-L) Whole-mount images (dorsal up, anterior left) showing dorsolateral views of the otic vesicle (outlined) in control embryos, *tfap2a* ^-/-^ mutants and *tfap2a* morphants, and *hs*:*tfap2a* embryos for the indicated genes at 26 hpf. (M) Mean and standard deviation of the total number of hair cells in utricular and saccular maculae of control and *hs*:*tfap2a* embryos and *tfap2a* morphants at the indicated times (n = 24 embryos each). Data were obtained by counting GFP-positive hair cells in the sensory epithelia of *brn3c*:*Gfp* transgenic embryos. Accumulation of hair cells was normal except in *tfap2a* morphants at 36 hpf (*), which showed a small but significant decrease relative to the control.

To determine whether the above changes in SAG development resulted from mis-patterning of the otic vesicle, we examined expression of several regional markers. In *tfap2a* ^*-/-*^ mutants and *tfap2a* morphants, domains of the dorsal marker *dlx3b* and the ventrolateral marker *otx1b* were slightly contracted (Fig. 5B, E). Domains of *pax5*, an anterior-ventral marker of the utricular macula, and *pou3f3b*, a posterior-medial marker of the saccular macula were not altered (Fig. 5H, K). Additionally, sensory epithelia appeared to develop normally and there were no obvious changes in hair cell development through 54 hpf (Fig. 5M). Activation of *hs*:*tfap2a* at 24 hpf led to weak contraction *otx1b* but a substantial expansion of the *dlx3b* (Fig. 5C, F). *pax5* was expressed in its normal domain but at a reduced level (Fig. 5L). Expression of *pou3f3b* was normal (Fig. 5I) and there were no changes in accumulation of hair cells through 54 hpf (Fig. 5M). Thus, gross patterning in the otic vesicle was nearly normal in *tfap2a* ^*-/-*^ mutants and morphants, though substantial changes were seen in one marker (*dlx3b*) following overexpression of *tfap2a*. Such changes in gene expression likely reflect changes in cell signaling as described below.

### Effects on Notch and Fgf signaling

**Fig 6:**
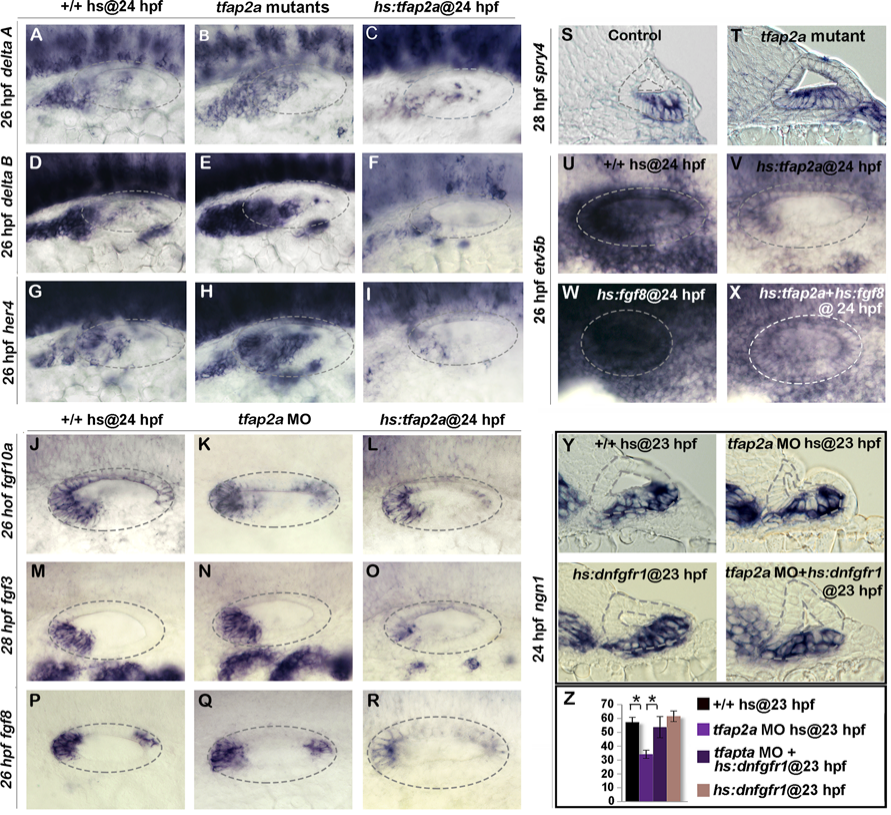
Tfap2a regulates the level of Fgf and Notch Signaling in the otic vesicle. (A-V) Whole-mount images (dorsal up, anterior left) showing dorsolateral views of the otic vesicle (outlined). (A-R) Expression of the indicated genes in wild-type embryos, *hs*:*tfap2a* embryos and *tfap2a* ^-/-^ mutants (A-I) or *tfap2a* morphants (J-R) at 26 hpf (A-L, P-R) and 28 (M-O) hpf. (S, T) Cross-sections (dorsal up, medial left) passing through the utricular macula show *spry4* expression at 28 hpf in a control embryo and *tfap2a* ^-/-^ mutant. (U-X) Whole-mounts showing expression of *etv5b* at 26 hpf. Activation of *hs*:*tfap2a* diminishes *etv5b* expression (U, V), activation of *hs*:*fgf8* leads to global upregulation of *etv5b* (W), and co-activation of *hs*:*fgf8* and *hs*:*tfap2a* restores *etv5b* to near normal (X). (Y) Cross-sections (dorsal up, medial left) passing just posterior to the utricular macula showing *ngn1* at 24 hpf following a 35°C heat shock at 23 hpf. Reduction in the *ngn1* domain caused by knockdown of *tfap2a* is rescued by weak activation of *hs*:*dnfgfr1*. (Z) Mean and standard deviation of the total number of *ngn1* positive cells in the otic epithelium at 24 hpf for the genotypes and knockdowns indicated in the color key (counted from serial sections, n = 3–6 ears per time point). Asterisks (*) indicate statistically significant differences between the groups indicated in brackets.

We next investigated whether Tfap2a activity influences Notch and Fgf signaling, pathways known to regulate development of SAG neurons. For example, Delta-Notch signaling is normally activated by neurogenic factors Ngn1 and Neurod and serves as a feedback inhibitor of neurogenesis [2,33,34]. Disruption of Delta-Notch signaling leads to excess neural specification and precocious differentiation [35], similar to the effects of misexpression of *tfap2a*. Here we observed that expression of *deltaA* and *deltaB* was increased in *tfap2a* ^*-/-*^ mutants and, conversely, *delta* gene expression was strongly impaired following overexpression of *tfap2a* (Fig. 6A-F). Similar changes were observed for the Notch target gene *her4*, which increased in *tfap2a* ^*-/-*^ mutants and decreased following activation of *hs*:*tfap2a* (Fig. 6G-I). Thus Tfap2a appears to inhibit Notch activity during development of SAG neurons by inhibiting expression of Notch ligands.

Fgf signaling has a more complex role in neural development in the ear. At early stages specification of neuroblasts requires Fgf. As development proceeds, however, rising levels of Fgf5 secreted by mature SAG neurons terminates specification of new neuroblasts and delays differentiation of TA cells into mature neurons [5] (Fig. 1K). Weak impairment of Fgf signaling can prolong neurogenesis and accelerate neural differentiation, mimicking aspects of the *tfap2a* overexpression phenotype. We therefore examined expression of various *fgf* genes in the otic vesicle. Knockdown of *tfap2a* did not appear to alter expression of *fgf3*, *fgf8*, or *fgf10a* (Fig. 6K, N, Q). Activation of *hs*:*tfap2a* at 24 hpf led to reduced expression of *fgf3* and *fgf8*, but expression of *fgf10a* was not altered (Fig. 6L, O, R). To look for changes in Fgf signaling, we examined expression of Fgf-target genes *spry4*, *etv4* (*pea3*) and *etv5b* (*erm*). Although *etv4* and *etv5b* expression appeared normal in *tfap2a* morphants (S3D, H Fig.), *spry4* was expressed at higher levels and in a broader domain than normal in *tfap2a* ^*-/-*^ mutants, indicating that Fgf signaling was elevated (Fig. 6S-T). Conversely, activation of *hs*:*tfap2a* at 24 hpf reduced expression of *etv4*, *etv5b* and *spry4* by 26 hpf, indicating a reduced level of Fgf signaling (Fig. 6U-V, S3A-J Fig.). Fgf signaling remained reduced through 28 hpf but started to recover after 30 hpf as transgene activity decayed (S3K-Q Fig.). Thus, Tfap2a appears to limit Fgf signaling, in part through reducing expression of *fgf* genes.

To test whether Tfap2a can influence Fgf signaling independently of ligand expression, we co-misexpressed *tfap2a* and *fgf8*. While activation of *hs*:*fgf8* led to global upregulation of *etv5b*, co-activation of *hs*:*tfap2a* with *hs*:*fgf8* at 24 hpf partially suppressed expression of *etv5b* (Fig. 6W-X). This indicates that *tfap2a* can inhibit Fgf signaling at a level downstream of ligand accumulation.

To test the functional significance of elevated Fgf signaling in *tfap2a* morphants, we examined whether weakly inhibiting Fgf signaling could rescue the neurogenic deficiencies in *tfap2a* morphants. Indeed, reducing the level of Fgf signaling via low-level activation of *hs*:*dnfgfr1* (dominant-negative Fgf receptor) at 35°C restored *ngn1+* cell counts to normal in *tfap2a* morphants (Fig. 6Y-Z). This suggests that elevated Fgf signaling partially accounts for the reduced neuroblast specification in *tfap2a* morphants and mutants.

**Fig 7:**
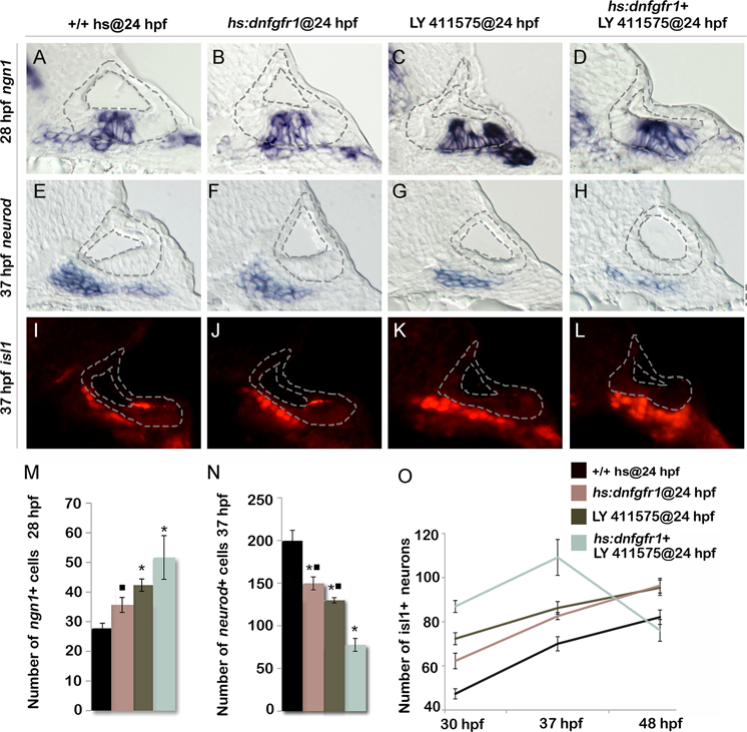
Reducing Fgf and Notch levels mimics the effects of *tfap2a* overexpression. (A-L) Cross-sections (medial left, dorsal up) passing just posterior to the utricular macula and showing expression of *ngn1* at 28 hpf (A-D), or sections passing through the utricular macula and showing *neurod* at 37 hpf (E-H) or Isl1 at 37 hpf (I-L) in wild-type control (A, E, I), *hs*:*dnfgfr1* embryos (B, F, J), LY411575 inhibitor treated wild-type embryos (C, G, K) and LY411575 inhibitor treated *hs*:*dnfgfr1* embryos (D, H, L). All specimens were treated with 0.3% DMSO and heat-shocked at 24 hpf. The otic vesicle is outlined in each image. (M, N) Mean and standard deviation of the total number of *ngn1* positive cells in the otic epithelium at 28 hpf (M) and total *neurod* positive SAG precursors at 37 hpf (N) for the genotypes and treatments indicated in the color key (counted from serial sections, n = 3–6 ears per time point). Asterisks (*) indicate significant differences from control embryos and filled squares indicate significant differences relative to *hs*:*dnfgfr1* embryos treated with LY411575. (O) Mean and standard deviation of the total number of Isl1 positive SAG neurons at different times for the genotypes and treatments indicated in the color key (n = 6–15 embryos each). In (O) differences between control and experimental specimens were significant at each time point. In addition, LY411575 treated *hs*:*dnfgfr1* embryos were significantly different from *hs*:*dnfgfr1* alone or LY411575 treatment alone.

Because Tfap2a appears to dampen both Fgf and Notch signaling, we tested whether weakening both pathways by other means could mimic the effects of *tfap2a* overexpression. Low level activation of *hs*:*dnfgfr1* at 36.5°C increased the number of *ngn1*+ neuroblasts in the ear by ~30% (Fig. 7B, M). Similarly, reducing the level of Notch signaling by treatment with the gamma-secretase inhibitor LY411575 increased the number of *ngn1+* cells in the otic vesicle by ~50% (Fig. 7C, M). Combining these conditions to reduce both Fgf and Notch signaling further increased neuroblast specification, closely mimicking the effects of activating *hs*:*tfap2a* at 24 hpf (Fig. 7D, M). Likewise, inhibiting both Fgf and Notch together reduced the number of *neurod*+ cells in the TA pool and increased accumulation of Isl1+ neurons through 37 hpf in a manner similar to activating *hs*:*tfap2a* at 24 hpf (Fig. 7E-O). Moreover, embryos inhibited for both Fgf and Notch signaling showed a dramatic loss of mature SAG neurons after 37 hpf, again mimicking the effects of *hs*:*tfap2a* activation (Fig. 7O). Interestingly, inhibition of either Fgf or Notch alone caused similar but more modest acceleration of neural differentiation, but such conditions did not lead to subsequent loss of mature neurons after 37 hpf (Fig. 7O). This is possibly because differentiation, though accelerated relative to control embryos, is still slow enough to allow expression of all factors essential for survival. Finally, activating *hs*:*tfap2a* at 24 hpf combined with conditions to inhibit Fgf and Notch did not further increase accumulation of Isl1+ neurons at 37 hpf (S4 Fig.). Thus, reducing both Fgf and Notch signaling is sufficient to recapitulate all observed effects of *tfap2a* overexpression.

### Tfap2a regulates transit amplification independently of earlier stages

**Fig 8:**
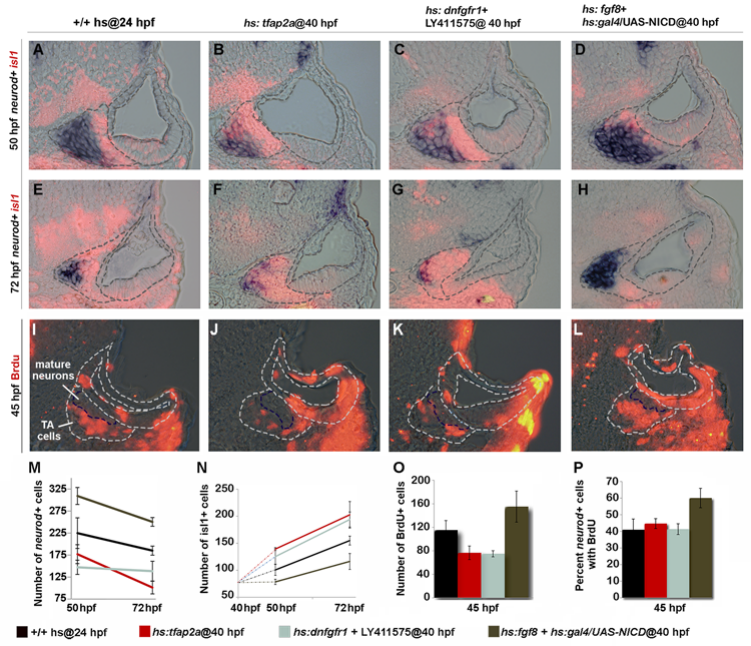
Tfap2a regulates development of TA cells independent of earlier stages. (A-L) Cross-sections (medial left, dorsal up) at the level of utricular macula. The otic vesicle and regions occupied by mature SAG neurons and TA cells are outlined in each image. Co-staining for Isl1 (red) and *neurod* (blue) at 50 hpf (A-D) and 72 hpf (E-H) under conditions indicated across the top of the figure. All specimens were treated with 0.3% DMSO and heat-shocked (39°C) at 40 hpf. (I-L) BrdU staining in embryos exposed to BrdU for 5 hours, from 40 to 45 hpf. The region containing mature SAG neurons is indicated. (M-O) Mean and standard deviation for the total number of *neurod*+ TA cells (M), total Isl1+ mature neurons (N) and total BrdU+ TA cells (O) for the indicated genotypes and treatments (counted from serial sections, n = 3–6 ears per time point). Differences between control and experimental specimens were significant at all time points, except that *hs*:*fgf8*+*hs*:*gal4*/*UAS-NICD* embryos were not significantly different from control embryos at 72 hpf in (N). (P) Percentage of *neurod*+ TA cells that are also BrdU positive (counted from serial sections, n = 3–6 ears per time point). Only *hs*:*fgf8*+*hs*:*gal4*/*UAS-NICD* embryos were significantly different from the control in (P).

We considered the possibility that the ability of Tfap2a to alter development of SAG cells outside the ear could arise secondarily from perturbation of earlier developmental stages within the otic vesicle. To test whether Tfap2a can specifically influence cells after delamination (without altering early development within the otic vesicle), we activated *hs*:*tfap2a* at 40 hpf when neurogenesis has ceased in the otic vesicle. Recall that transgene activation fails to prolong or reinitiate neuroblast specification at this stage (Fig. 2L). Regardless, overexpression of *tfap2a* at 40 hpf still reduced the number of *neurod*+ cells and led to an increase in Isl1+ neurons at 50 hpf (Fig. 8B, F, M, N). Misexpression of *tfap2a* also reduced the total number of cells in the TA pool that incorporated BrdU (Fig. 8J, O). However, this was proportional to the reduction in the total number of *neurod*+ cells (Fig. 8P), consistent with acceleration of the entire TA pool. A notable difference from earlier activation is that activating *hs*:*tfap2a* at 40 hpf did not lead to apoptosis of SAG neurons at later stages, as the number of Isl1+ cells remained elevated and in fact continued to increase through at least 72 hpf (Fig. 8B, F, N). The same effects were obtained by directly inhibiting both Fgf and Notch signaling after 40 hpf (Fig. 8C, G, K M-P). Conversely, misexpressing Fgf8 and NICD (Notch intracellular domain) by heat shock activation at 40 hpf led to accumulation of more TA cells and fewer mature neurons than normal at 50 and 72 hpf, indicating a delay in neuronal differentiation (Fig. 8D, H, M, N). Moreover, activating Fgf and Notch together increased the percentage of *neurod*+ cells that continue to incorporate BrdU (Fig. 8L, O, P), suggesting that the majority of cells in the TA pool persist in a relatively immature stage of SAG development. Thus, manipulating *tfap2a*, or Fgf and Notch signaling directly, can alter the rate of differentiation of TA cells even when earlier development within the otic vesicle has occurred normally. On the other hand, survival of mature SAG neurons requires normal development at early stages.

### Tfap2a acts non-autonomously

**Fig 9:**
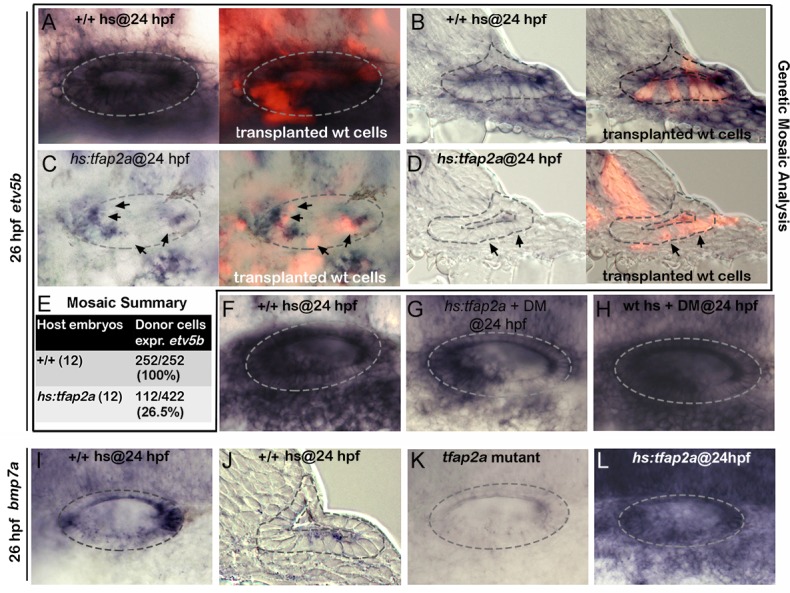
Tfap2a regulates SAG development non-autonomously. (A-D) Whole-mounts (dorsal up, anterior left) and cross-sections (just posterior to utricular macula, medial to the left) showing both bright-field and corresponding fluorescent images of +/+ host embryos (A, B) and *hs*:*tfap2a* host embryos stained for *etv5b* expression (blue) and showing transplanted wild-type donor cells (red). Positions of wild-type cells that fail to express *etv5b* are highlighted with arrows in (C, D). (E) Summary of mosaic analysis showing the number of +/+ or *hs*:*tfap2a* host embryos examined and the number of +/+ donor cells expressing *etv5b* over the total number of donor cells that populated the ventral half of the otic vesicle. (F-H) Expression of *etv5b* at 26 hpf in a control (F), a DM-treated *hs*:*tfap2a* embryo (G) and DM-treated wild-type embryo (H). All specimens were treated with 1% DMSO and heat-shocked at 24 hpf. (I-L) Expression of *bmp7a* at 26 hpf in a control (I, J), *tfap2a* ^*-/-*^ mutant (K) and *hs*:*tfap2a* embryo (L). Wild-type and *hs*:*tfap2a* embryos were heat shocked at 24 hpf.

It is noteworthy that *tfap2a* is not normally expressed in the TA pool or mature neurons, yet knockdown or misexpression of *tfap2a* alters the rate of differentiation and survival of these cells. This raised the possibility that Tfap2a could act non-autonomously on cells outside the ear. To test directly whether *tfap2a* can act non-autonomously, we generated genetic mosaics by transplanting wild-type cells into *hs*:*tfap2a* host embryos. We reasoned that if *hs*:*tfap2a* were to act non-autonomously, activating the transgene in host cells should be able to prevent wild-type cells from responding to Fgf sources in the otic vesicle. In support, activation of *hs*:*tfap2a* at 24 hpf suppressed *etv5b* expression in the majority (73.5%) of transplanted wild-type cells by 26 hpf (Fig. 9C-E), indicating that *tfap2a* acts non-autonomously to modulate the response to Fgf. In contrast, all wild-type donor cells transplanted into wild-type host embryos showed normal expression of *etv5b* in the otic vesicle (Fig. 9A, B, E). Thus Tfap2a non-autonomously inhibits Fgf signaling in the otic vesicle.

### Bmp7a mediates the effects of Tfap2a

Bmp signaling is well known to antagonize Fgf signaling in a variety of developmental contexts. Here we found that blocking Bmp signaling with the pharmacological inhibitor dorsomorphin (DM) [36] strongly suppressed the ability of *hs*:*tfap2a* to reduce Fgf signaling. For example, *etv5b* expression was nearly normal in the otic vesicle 2 hours after the activation of *hs*:*tfap2a* when embryos were also treated with DM (Fig. 9F, G). DM treatment alone had negligible effects on expression of *etv5b* (Fig. 9H). We next surveyed expression of various *bmp* genes following activation of *tfap2a* and identified *bmp7a* as a likely candidate for mediating its effects on Fgf signaling. *bmp7a* is normally expressed in cells at the anterior and posterior ends of the otic vesicle, and at a lower level in a ventrolateral domain that overlaps the *tfap2a* expression domain [37] (Fig. 9I-J, compare with Fig. 1C). Expression of *bmp7a* was nearly abolished in *tfap2a* ^*-/-*^ mutants (Fig. 9K). In contrast, activation of *hs*:*tfap2a* strongly upregulated *bmp7a* expression in the otic vesicle as well as in surrounding tissues (Fig. 9L). In contrast to *bmp7a*, we observed no consistent changes in expression of *bmp2b* or *bmp4* following manipulation of *tfap2a* function (S5 Fig.), indicating that changes in *bmp7a* are relatively specific. Together, these data suggest that *tfap2a* positively regulates *bmp7a*, which in turn restricts the level of Fgf signaling during otic development.

**Fig 10:**
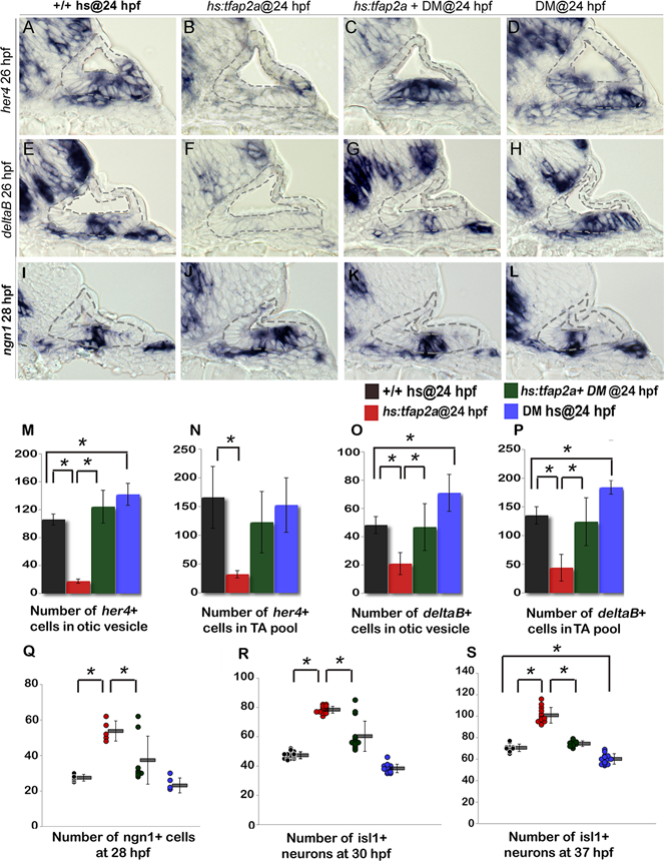
Bmp signaling mediates the effects of Tfap2a on SAG development. (A-L) Cross sections (medial left, dorsal up) through the otic vesicle just posterior to the utricular macula showing expression of *her4* (A-D) and *deltaB* (E-H) at 26 hpf and *ngn1* (I-L) at 28 hpf in control embryos (A, E, I), *hs*:*tfap2a* embryos (B, F, J), DM-treated *hs*:*tfap2a* embryos (C, G, K) and DM-treated wild-type embryos (D, H, L). All specimens were treated with 1% DMSO and heat-shocked at 24 hpf. (M-P) Mean and standard deviation of the total number of *deltaB* or *her4* expressing cells inside the otic vesicle or in the TA pool at 26 hpf under conditions indicated in the color key (counted from serial sections, n = 3–6 ears per time point). (Q-S) Mean and standard deviation of the total number of *ngn1*+ cells at 28 hpf (Q) and Isl1+ SAG neurons at 30 hpf (R) and 37 hpf (S) under the conditions indicated in the color key. Asterisks (*) indicate statistical differences between the groups indicated in brackets.

We next examined whether Bmp signaling mediates the effects of *tfap2a* on Notch activity. In support, blocking Bmp with DM rescued expression of *deltaB* and *her4* following activation of *hs*:*tfap2a* at 24 hpf (Fig. 10A-L, M-P). Treatment with DM alone caused a slight but significant increase in the number of cells expressing *deltaB* and *her4* (Fig. 10D, H, I-L). Thus the ability of *tfap2a* to restrict Notch signaling requires elevated Bmp signaling.

Finally, we tested whether the effects of *tfap2a* on SAG development also require Bmp signaling. Blocking Bmp after activating *hs*:*tfap2a* at 24 hpf restored neuroblast specification to normal in 6 out of 8 specimens (Fig. 10I-L, Q). Similarly, accumulation of mature Isl1+ neurons was nearly normal in 12 out of 15 embryos at 30 hpf (Fig. 10R) and was restored to normal in all specimens at 37 hpf (n = 15) (Fig. 10S). DM treatment alone caused a slight but significant reduction in neuroblast specification and accumulation of mature SAG neurons (Fig. 10Q-S). Together, these findings support a model in which *tfap2a* regulates the level of *bmp7a* expression in the otic vesicle, which in turn restricts Fgf and Notch signaling to control the amount, duration and speed of SAG development.

## Discussion

We have shown that Tfap2a regulates development of SAG neurons by modulating Fgf, Notch and Bmp signaling. Tfap2a overexpression promotes neurogenic specification in the otic vesicle and accelerates subsequent differentiation of TA precursors into mature neurons. Conversely, disruption of *tfap2a* reduces the number of *ngn1*+ neuroblasts and decreases the rate of neuroblast differentiation. The neurogenic effects of *tfap2a* overexpression result from inhibition of Notch and Fgf signaling, which normally serve to restrict neuroblast specification and delay differentiation. The effects of Tfap2a appear to be mediated by Bmp7a, which is upregulated in response to Tfap2a activity. Bmp signaling in turn antagonizes Fgf and Notch signaling to promote specification and terminal differentiation of SAG precursors. These findings are novel and clarify several aspects of SAG development, which are discussed further below.

### Coordination of Fgf and Notch

The interplay between Fgf and Notch is complex and dynamic, and levels must be precisely balanced for proper development of the SAG. A low-to-moderate level of Fgf is required to initiate neurogenesis by activating expression of *ngn1* [5,7,38–40], which in turn activates expression of Notch ligands [2,34]. Notch activity serves to limit and slow neurogenesis [33,34]. Neurogenesis is also inhibited at later stages by rising levels of Fgf5 derived from mature neurons [5] (Fig. 1K). Without proper modulation by *tfap2a*, both Fgf and Notch signaling quickly become overactive, which terminates specification prematurely and impedes maturation of neuroblasts in the TA pool, thereby leading to under-production of mature SAG neurons.

While too much Fgf and Notch activity clearly impairs neurogenesis, insufficient levels are ultimately far more damaging to SAG development. Conditions that reduce Fgf and Notch signaling (e.g. overexpression of *tfap2a*) cause dramatic acceleration of differentiation and overproduction of neurons, most of which later die upon maturation. Neuronal death appears to arise secondarily from acceleration of early stages within the otic vesicle, as accelerating later stages does not lead to neuronal death. It is likely that early neuroblast differentiation is especially sensitive to acceleration due to insufficient buildup of factors needed for survival and function of mature neurons. In contrast, an important attribute of the TA pool is that the rate of differentiation can be regulated without compromising subsequent neuronal survival. The TA pool represents a relatively stable population of slowly cycling progenitors that must be maintained to meet the needs of the growing larva or to regenerate new neurons following damage [5]. It is likely that *tfap2a* is needed only transiently in the otic vesicle to prolong specification and establish a healthy progenitor population. Once neuroblast specification has terminated, however, downregulation of *tfap2a* appears necessary to allow Fgf and Notch signaling to rise sufficiently to balance rates of proliferation vs. differentiation in the TA pool.

### The role of Bmp

We have shown for the first time a role for Bmp in SAG development. Numerous earlier studies have shown that Bmp regulates morphogenesis of semicircular canals [41–44] and numerous aspects of development of sensory epithelia [45–49]. Bmp has also been found to promote SAG survival and neurite outgrowth in chick explant cultures [50]. However, no previous studies have detected a role in specification or differentiation of SAG neurons. Abello et al. [38] reported that blocking Bmp signaling did not alter the size of the neurogenic domain in the chick otic vesicle; and mosaic misexpression of an activated form of Bmp receptor did not inhibit neurogenesis, though the possibility that it may have accelerated neurogenesis was not examined. In comparison, we find that *tfap2a* activates expression of *bmp7a* and that blocking Bmp signaling reverses the effects of *tfap2a* overexpression. Treating wild-type embryos with DM partially mimics the effects of disrupting *tfap2a*. The more severe defects caused by *tfap2a* loss of function could indicate that additional factors help mediate the effects of Tfap2a. Alternatively, Bmp7a could act partly through non-canonical signaling, similar to the role of Bmp7 in establishing tonotopy in the organ of Corti in mouse [46]. The specific requirement for Bmp7a in SAG development cannot be assessed by examining *bmp7a* mutants because development of the otic placode is severely compromised [51]. Development of lines to conditionally disrupt or misexpress *bmp7a* could resolve many of these issues.

Interestingly, in zebrafish Bmp effectors Smad1 and Smad5 are specifically upregulated in delaminated SAG cells [37,52] (Fig. 1K). This constitutes an unusual form of regulation because *smad1/5* genes are broadly expressed with relatively little variation in the level of expression. Elevated levels of Smad1/5 accumulation could render SAG precursors outside the ear especially sensitive to Bmp, explaining how Bmp expressed within the otic vesicle could have such a profound effect on development of TA cells.

### Regulation of *tfap2a*

We do not yet know how *tfap2a* is regulated in the otic vesicle. We detect no changes in expression after manipulating levels of Fgf, Notch, Bmp, Wnt or *ngn1*. It is possible that expression of *tfap2a* in the otic placode and otic vesicle reflects auto-regulatory maintenance from earlier stages. During gastrulation *tfap2a* is induced by Bmp in non-neural ectoderm where it functions as a competence factor for preplacodal development [9]. Once induced Tfap2a acts to maintain its own expression even if Bmp is subsequently blocked [11]. This is an important aspect of regulation because dorsally expressed Bmp-antagonists are required to initiate preplacodal development near the end of gastrulation [9,53–55]. Although expression of *tfap2a* could simply persist in the otic placode through self-maintenance, it is not clear how expression becomes restricted to ventrolateral cells in the otic vesicle. A similar pattern is seen in the chick otic vesicle, possibly indicating a conserved mechanism ([56]; Fig. 1). Identifying factors that regulate *tfap2a* in zebrafish and chick will likely shed light on general mechanisms of otic patterning and specific mechanisms of otic neurogenesis.

## Materials and Methods

### Fish strains and developmental conditions

The wild type strains were derived from AB line (Eugene, OR). Transgenic lines used in this study include *Tg(hsp70*:*tfap2a)* ^*x24*^ [11], *Tg(hsp70*:*fgf8a)* ^*x17*^ [57], *Tg(hsp70I*:*dnfgfr1-EGFP)* ^*pd1*^ [58], *Tg(hsp70I*:*gal41*.*5*)^*kca4*^ [59], *Tg(UAS*:*myc-Notch1a-intra)* ^*kca3*^ [60] and *Tg(brn3c*:*gap43-GFP)* ^*s356t*^ [61]. These transgenic lines are referred as *hs*:*tfap2a*, *hs*:*fgf8*, *hs*:*dnfgfr1*, *hs*:*gal4/UAS-NICD* and *brn3c-GFP* respectively. Mutant line *tfap2a* ^*m819*^ [62] was used for most loss of function studies. Mutants were identified by characteristic phenotypes showing expected Mendelian frequencies. Except where noted, embryos were maintained at 28.5°C in fish water containing 0.008% Instant Ocean salts, methylene blue and PTU (1-phenyl 2-thiourea, 0.3 mg/ml, Sigma) to block melanin formation. Embryos were staged according to standard protocols [63].

### Gene misexpression and morpholino injection

To activate heat-shock inducible transgenic lines, heterozygous transgenic embryos were incubated at 39°C for 30 minutes except where noted. Under this condition, activation of *hs*:*tfap2a* at 24 hpf led to a detectable increase in *tfap2a* levels by the end of the heat-shock period. Maximal *tfap2a* expression was seen at 25.5 hpf and the *tfap2a* levels remained elevated in the otic vesicle thorough at least 29 hpf (S1 Fig.). Since the complete blockage of Fgf signaling inhibits neurogenic specification in the otic vesicle [5], weak attenuation of Fgf signaling was achieved by activation of *hs*:*dnfgfr1* at 35°C for 30 minutes (Fig. 6) or at 36.5°C for 30 minutes (Fig. 7). After the heat-shock, embryos were incubated at 33°C until fixation. In some loss of function experiments, *tfap2a* was knocked down by injecting embryos at the 1-cell stage with approximately 5 ng of *tfap2a* morpholino oligomer (MO). The sequence of *tfap2a* MO has been tested previously for specificity and efficiency [64].

### Pharmacological treatments

Notch signaling was blocked by treating embryos with LY411575 diluted from a 10 mM stock in DMSO to a final concentration of 30 μM in fish water. Bmp signaling was blocked by Dorsomorphin (Sigma, P5499) diluted from a 10 mM stock solution into a final concentration of 100 μM in fish water. Treatments were carried in a 24-well plate with a maximum of 15 embryos per well in a volume of 500 μl each.

### *In situ* hybridization and immunohistochemistry

In situ hybridization and antibody staining was carried out as described previously [65–67]. In situ TUNEL assay was performed by using Promega terminal deoxynucleotidyl transferase (M828A) according to the manufacturer’s protocol. Following primary and secondary antibodies were used in this study: anti-Islet1/2 (Developmental Studies Hybridoma Bank 39.4D5, 1:100 for whole-mount, 1:250 for cryo-sections), anti-BrdU (Beckton-Dickinson, 1:250) and Alexa 546 goat anti-mouse IgG (Invitrogen A-11003, 1:50 for whole-mount, 1:250 for cryo-sections). Cryo-sectioning and BrdU labeling were carried out as described previously [5]. Whole-mount stained embryos were sectioned except for Figs. 1, 7 and S4 where anti-islet1/2 staining was performed on sections using standard whole mount protocols.

### Cell transplantation

Wild-type donor cells were injected with the lineage tracer (tetramethylrhodamine labeled, 10,000 MW, lysine-fixable dextran in 0.2 M KCl) and transplanted into non-labeled *hs*:*tfap2a* embryos at blastula stage.

### Chick experiments

Embryonic day 3 (E3) and E4 chick embryos were fixed and embedded in gelatin (7.5% gelatin, 15% sucrose in PBS). 14μm thick sections were collected on Superfrost Plus slides. For AP2a and Jagged-1 co-detection, slides were boiled in 10mM citric acid for 10 minutes prior to antibody application and then incubated in 0.012% hydrogen peroxide for 15 minutes at room temperature. The 3B5 AP2α monoclonal antibody developed by Trevor Williams was obtained from the Developmental Studies Hybridoma Bank developed under the auspices of the NICHD and maintained by the University of Iowa, Department of Biology, Iowa City, IA 52242. AP2α antibody was diluted 1:100 and Jagged-1 polyclonal antibody (Santa Cruz Biotechnology H-114) was diluted 1:200 in blocking buffer (PBS with 0.02% Tween-20, 0.1% Triton X-100, and 10% goat serum). Staining was detected with biotinylated mouse secondary antibody (Mouse Vectastain ABC kit) in conjunction with PerkinElmer TSA Plus Cyanine-3 System and AlexaFluor 488 conjugated rabbit secondary antibody diluted 1:500 in A+B substrate solution (AlexaFluor goat anti-rabbit, Invitrogen). All slides were mounted in Fluoromount G (Southern Biotech).

### Statistics

For pairwise comparisons, student’s t-tests were used to evaluate significance. For experiments involving more than two groups significance was evaluated using one-way ANOVA and Tukey post-hoc HSD tests.

### Ethics statement

The studies described herein were fully compliant with federal guidelines and IACUC-approved Animal Use Protocol number 2012–011.

## Supporting Information

S1 Fig Heat-shock activation of *hs*:*tfap2a* transgene leads to transient misexpression of *tfap2a*. (A-L) Whole-mount images (dorsal up, anterior left) showing *tfap2a* expression in wild-type and *hs*:*tfap2a* embryos. Embryos were fixed and stained at indicated intervals after the end of a 30-minute heat-shock initiated at 24 hpf.
(TIF)

S2 Fig Maturation of SAG neurons remains deficient in *tfap2a* mutants at 3 dpf. (A-F) Cross-sections (dorsal up, medial left) pass through the anterior (A, B), middle (C, D), and posterior (E, F) parts of the otic vesicle and show *isl1* staining in a wild-type embryo (A, C, E) and a *tfap2a* mutant (B, D, F) embryo at 72 hpf. (G) Mean and standard deviation of the total number *Isl1+* SAG neurons in wild-type (n = 3) and *tfap2a* mutant (n = 4) embryos at 72 hpf (counted on serial sections). Asterisk (*) indicate statistically significant difference compared to wild-type embryos.
(TIF)

S3 Fig *tfap2a* inhibits Fgf signaling in the otic vesicle. (A-J): Cross-sections (dorsal up, medial left) passing through the otic vesicle just posterior to the utricle showing expression of *etv4* (A-D), *etv5b* (E-H) and *sprouty4* (I, J) in heat-shocked wild-type (A, E, I), *hs*:*tfap2a* (B, F, J), non-heat shocked wild-type (C,G) and *tfap2a* morphant (D,H) embryos at indicated time points. (K-P): Whole-mount images (dorsal up, anterior left) showing dorsolateral views of the otic vesicle (outlined) stained for *etv5b* expression in heat-shocked wild-type and *hs*:*tfap2a* embryos at indicated times.
(TIF)

S4 Fig Inhibition of Notch and Fgf signaling in *hs*:*tfap2a* embryos does not enhance the effects of *hs*:*tfap2a* activation. (A-I): Cross-sections at the level of utricular macula (medial to the left, dorsal up) show bright field (A, D, G), fluorescent (B, E, H) and merged (C, F, I) images for *neurod* (blue) and *isl1* (red) in heat-shocked wild-type, *hs*:*tfap2a* and LY 411575 treated *hs*:*tfap2a+ hs*:*dnfgfr1* embryos at 37 hpf. All specimens were treated with 0.3% DMSO and heat-shocked (39°C, 30 minutes) at 24 hpf. (J) Mean and standard deviation of the total number of *is1*+ neurons at 37 hpf under the conditions indicated in the color key (n = 10–15 specimens each). (K) Mean and standard deviation of the total number of *nrd*+ neuroblasts at 37 hpf under the conditions indicated in the color key (n = 3–6 ears each, counted from serial sections). Both experimental conditions were significantly different compared to controls. n.s., no statistical difference between the groups indicated in brackets.
(TIF)

S5 Fig The effects of *tfap2a* overexpression and knock-down on *bmp2b* and *bmp4* expression. (A-H): Whole-mount images (dorsal up, anterior left) showing dorsolateral view of the otic vesicle (outlined) for *bmp2b* (A-D) and *bmp4* (E-H) expression for the indicated genotypes and conditions. Activation of *hs*:*tfap2a* appears to reduce expression of both genes in portions of the otic vesicle, but *bmb2b* is upregulated in the hindbrain (B) and *bmp4* is upregulated in the dorsal part of the otic vesicle (F). Knocking down *tfap2a* had little or no effect on either gene (D, H).
(TIF)
