# Supplementary figures and images for: Tfap2a Promotes Specification and Maturation of Neurons in the Inner Ear through Modulation of Bmp, Fgf and Notch Signaling
Source: PLoS Genet. 2015 Mar 17;11(3):e1005037. doi: 10.1371/journal.pgen.1005037 (PMC4364372; doi:10.1371/journal.pgen.1005037)

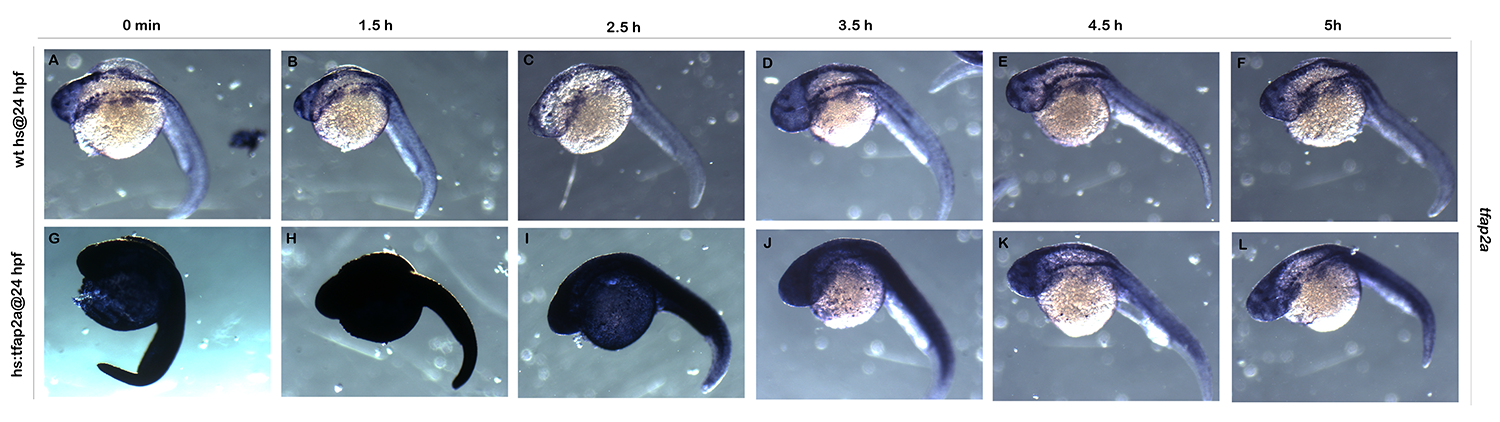

Supplement: S1 Fig — (A-L) Whole-mount images (dorsal up, anterior left) showing tfap2a expression in wild-type and hs:tfap2a embryos. Embryos were fixed and stained at indicated intervals after the end of a 30-minute heat-shock initiated at 24 hpf. (TIF) [file pgen.1005037.s001.tif]

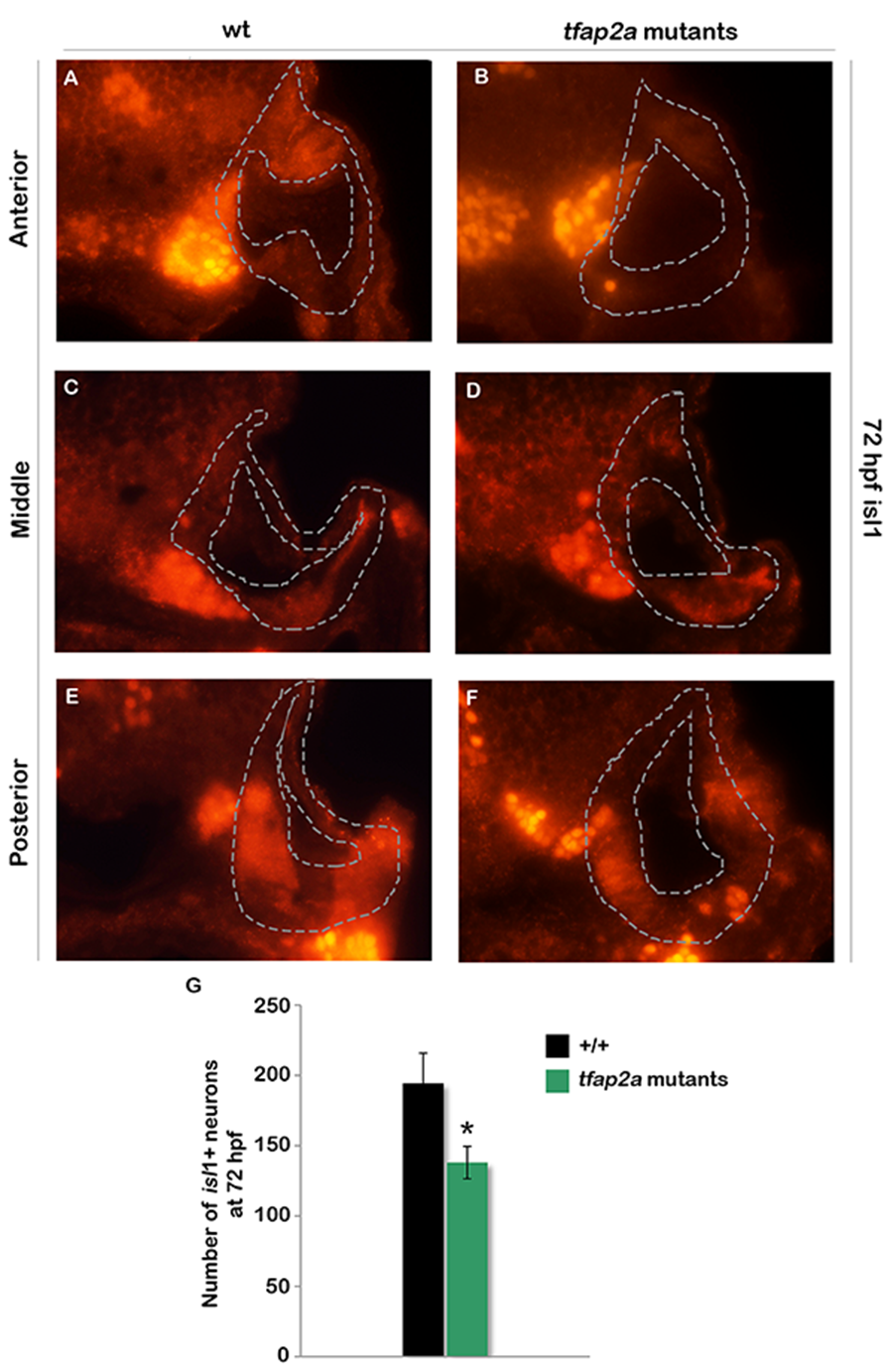

Supplement: S2 Fig — (A-F) Cross-sections (dorsal up, medial left) pass through the anterior (A, B), middle (C, D), and posterior (E, F) parts of the otic vesicle and show isl1 staining in a wild-type embryo (A, C, E) and a tfap2a mutant (B, D, F) embryo at 72 hpf. (G) Mean and standard deviation of the total number Isl1+ SAG neurons in wild-type (n = 3) and tfap2a mutant (n = 4) embryos at 72 hpf (counted on serial sections). Asterisk (*) indicate statistically significant difference compared to wild-type embryos. (TIF) [file pgen.1005037.s002.tif]

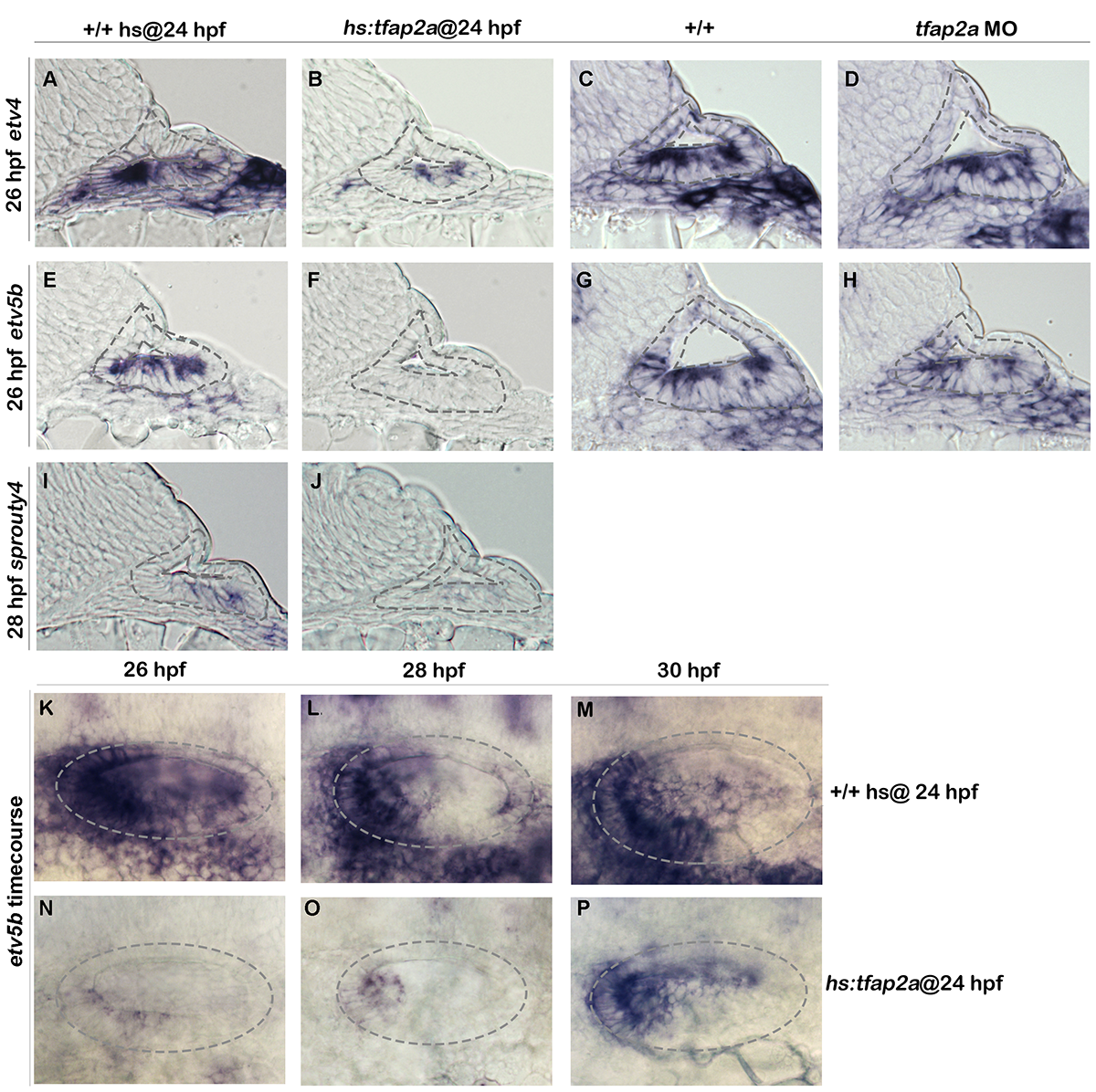

Supplement: S3 Fig — (A-J): Cross-sections (dorsal up, medial left) passing through the otic vesicle just posterior to the utricle showing expression of etv4 (A-D), etv5b (E-H) and sprouty4 (I, J) in heat-shocked wild-type (A, E, I), hs:tfap2a (B, F, J), non-heat shocked wild-type (C,G) and tfap2a morphant (D,H) embryos at indicated time points. (K-P): Whole-mount images (dorsal up, anterior left) showing dorsolateral views of the otic vesicle (outlined) stained for etv5b expression in heat-shocked wild-type and hs:tfap2a embryos at indicated times. (TIF) [file pgen.1005037.s003.tif]

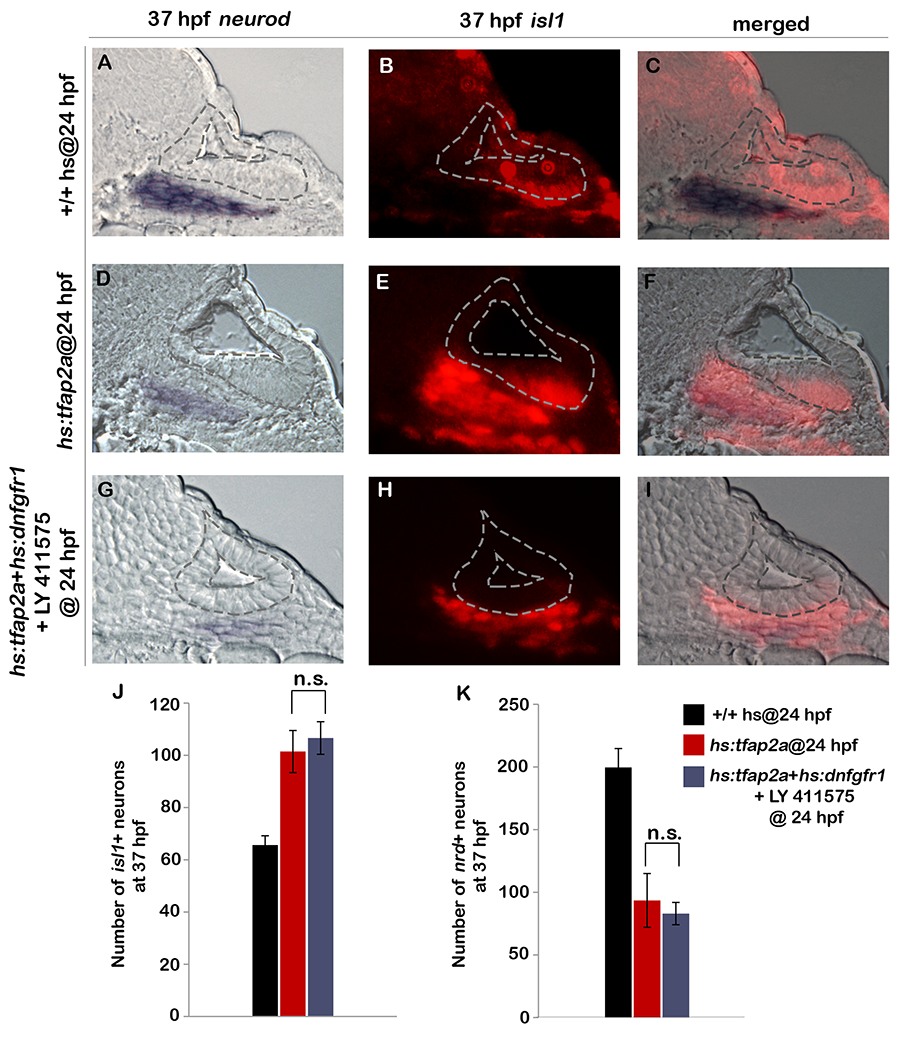

Supplement: S4 Fig — (A-I): Cross-sections at the level of utricular macula (medial to the left, dorsal up) show bright field (A, D, G), fluorescent (B, E, H) and merged (C, F, I) images for neurod (blue) and isl1 (red) in heat-shocked wild-type, hs:tfap2a and LY 411575 treated hs:tfap2a+ hs:dnfgfr1 embryos at 37 hpf. All specimens were treated with 0.3% DMSO and heat-shocked (39°C, 30 minutes) at 24 hpf. (J) Mean and standard deviation of the total number of is1+ neurons at 37 hpf under the conditions indicated in the color key (n = 10–15 specimens each). (K) Mean and standard deviation of the total number of nrd+ neuroblasts at 37 hpf under the conditions indicated in the color key (n = 3–6 ears each, counted from serial sections). Both experimental conditions were significantly different compared to controls. n.s., no statistical difference between the groups indicated in brackets. (TIF) [file pgen.1005037.s004.tif]

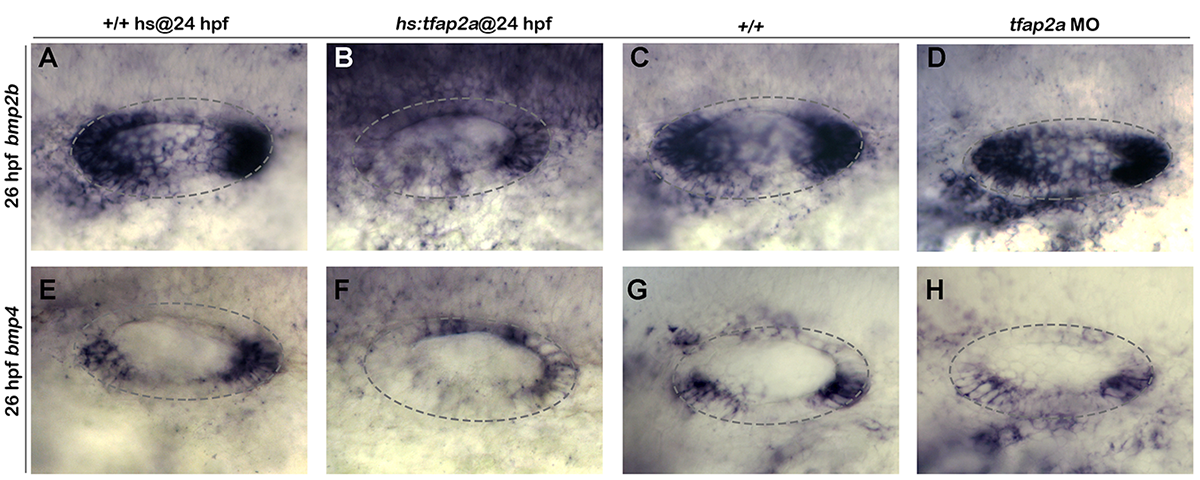

Supplement: S5 Fig — (A-H): Whole-mount images (dorsal up, anterior left) showing dorsolateral view of the otic vesicle (outlined) for bmp2b (A-D) and bmp4 (E-H) expression for the indicated genotypes and conditions. Activation of hs:tfap2a appears to reduce expression of both genes in portions of the otic vesicle, but bmb2b is upregulated in the hindbrain (B) and bmp4 is upregulated in the dorsal part of the otic vesicle (F). Knocking down tfap2a had little or no effect on either gene (D, H). (TIF) [file pgen.1005037.s005.tif]
